# Ars Longa, Vita Brevis

**DOI:** 10.3201/eid2903.AC2903

**Published:** 2023-03

**Authors:** Terence Chorba

**Affiliations:** Centers for Disease Control and Prevention, Atlanta, Georgia, USA

**Keywords:** endocarditis, tuberculosis, tuberculosis and other mycobacteria, bacteria, Mycobacterium tuberculosis, art science connection, emerging infectious diseases, art and medicine, about the cover, public health, ars longa, vita brevis, Frédéric François Chopin, Felix-Joseph Barrias, death of Chopin

**Figure:**
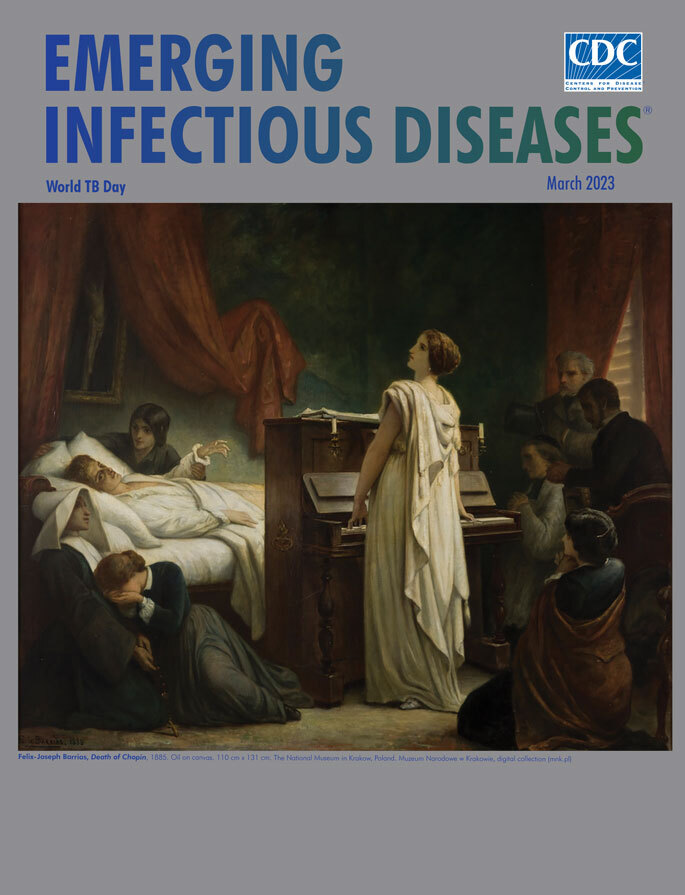
**Félix-Joseph Barrias (1822–1907). *Death of Chopin*, 1885,** Oil on canvas, 43.3 in × 51.6 in/110 cm × 131 cm. The National Museum in Krakow, Poland. Muzeum Narodowe w Krakowie, digital collection (https://mnk.pl).

“Life is short, art is long, opportunity is ephemeral...”

—Hippocrates, *Aphorisms*

Frédéric François Chopin (born Fryderyk Franciszek Chopin; 1810–1849) was a prolific Poland-born composer and pianist of outstanding technical ability and talent. Although his public career was limited to 30 performances, his contribution as a composer of significant works for piano is unparalleled in its vast array of genres, including ballades, études, impromptus, mazurkas, nocturnes, polonaises, préludes, scherzi, sonatas, and waltzes. All his works included piano and, although he wrote 2 piano concertos and some chamber music, most of his works were written for solo piano. 

Chopin was said to have been a sickly child; his younger sister Emilia died at the age of 14 from a rapidly progressive respiratory disease. Drawn by its identity as a center for the arts, he moved to Paris at age 21 and thrived as a master pianist and composer. Chopin continued in ill health throughout adulthood, suffering from shortness of breath, cervical lymphadenitis, night sweats, a persistent cough with copious sputum, and later hemoptysis. His physicians were reluctant to give a diagnosis of tuberculosis, a stigma-laden but probable diagnosis in the 19th century.

Without bacteriology or radiography, a definitive diagnosis of tuberculosis would not have been possible. It was not until March 1882 that Robert Koch announced his discovery of the causative organism of tuberculosis, *Mycobacterium tuberculosis*, and not until late 1895 that Wilhelm Roentgen developed the first radiograph. Thus, the definitive cause of Chopin’s death has remained speculative. Given that the composer’s sister had died with a similar respiratory affliction, a genetic condition has been proposed as an alternative, the most popular being cystic fibrosis, with its autosomal recessive inheritance pattern. Numerous other diagnoses considered have included aspergillosis, alpha-1 antitrypsin deficiency, granulomatosis with polyangiitis, hypogammaglobulinemia, idiopathic bronchiectasis, mitral stenosis, primary ciliary dyskinesia, tricuspid valve incompetence, pulmonary arteriovenous malformation, pulmonary hemosiderosis, and sarcoidosis, all of which may result in general weakness and nonspecific respiratory symptoms, such as dyspnea and chronic cough. 

In 1885, Félix-Joseph Barrias (1822‒1907) painted *Death of Chopin*, featured on this month’s cover. Barrias was a Paris native whose father was a successful painter on porcelain. Barrias learned the trade and its skills from his father but then became an illustrator and instructor in his own art school. Edgar Degas, a founder of Impressionism, was among Barrias’ many distinguished students. Like the works of his younger brother, Louis-Ernest Barrias, a well-renowned sculptor, most of Félix-Joseph Barrias’ known canvases have elements of Neoclassicism and Romanticism. Neoclassism is characterized as using the most attractive stylistic elements of the arts and culture of Graeco-Roman antiquity; Romanticism is characterized by emphasis on emotion, individualism, and idealization of heroic figures and their surroundings or environment, as in this portrayal of the death of the composer. 

When painting *Death of Chopin*, Barrias may have been inspired by a setting shortly before Chopin’s death, described vividly by Hungarian composer Franz Liszt, also a close friend of Chopin: “[Countess Potocka’s] tears were flowing fast when [Chopin] observed her standing at the foot of his bed, tall, slight, draped in white, resembling the beautiful angels created by the imagination of the most devout among the painters… He requested her to sing… The piano was rolled from his parlor to the door of his chamber, while, with sobs in her voice, and tears streaming down her cheeks, his gifted countrywoman sang…. He seemed to suffer less as he listened. She sang the famous Canticle to the Virgin [putatively from Stradella’s oratorio for St. John the Baptist (https://youtu.be/qO6i-0AbUGU)]… ‘How beautiful it is!’ he exclaimed. ‘My God, how very beautiful! Again – again!’... Chopin again feeling worse, everybody was seized with fright—by a spontaneous impulse all who were present threw themselves upon their knees—no one ventured to speak; the sacred silence was only broken by the voice of the Countess, floating, like a melody from heaven, above the sighs and sobs which formed its mournful earth accompaniment… A dying light lent its shadows to this sad scene. Chopin’s [older] sister [Ludwika] prostrated near his bed, wept, and prayed—and never quitted his attitude of supplication while the life of the brother she so cherished lasted.” Most, if not all, of those present would have known that Chopin had dedicated several enlivened, optimistic pieces to the singer including Waltz in D-flat major, Op. 64 (Minute Waltz; https://www.youtube.com/watch?v=3H0SRv8QNwk) and Prelude Op. 28, No 7 (https://www.youtube.com/watch?v=U6vJmHiHBMo), although the somber progressions of Prelude Op. 28, No. 4 (https://www.youtube.com/watch?v=Hj3daBN5F-o) would have been more appropriate to the moment. 

Near death, Chopin reportedly asked that his heart not be interred with his body but rather entombed in his native Poland. Ludwika commissioned an autopsy in which his heart was removed and put into a preservative liquid, probably brandy, which was often used for tissue preservation. 

Although the rest of Chopin’s remains were interred in Paris, Ludwika took the heart back to Poland, where it has remained in the preservative at the Church of the Holy Cross (*Bazylika Świętego Krzyża*) in Warsaw, except when briefly removed from the church under Nazi rule. In April 2014, a team of clergy, scientists, and 2 physicians—1 from the Polish Academy of Sciences’ Institute of Human Genetics and 1 from the Institute of Forensic Medicine at Wroclaw Medical University—were allowed to examine the composer’s heart visually, without opening its original jar of embalming liquid. Not surprisingly, their assessment was that Chopin had “a serious fibrinoid epicarditis embodied by foci of epicardial hyalinization in the left ventricular front wall and with dilatation, mainly of the right ventricle and right atrium (*cor pulmonale*) with pronounced features of chronic heart failure, predominantly of the right ventricle,” consistent with end-stage constrictive pericarditis with fibrosis, and “several glossy, whitish-pearl nodules, slightly protruding from the surface of the myocardium,” consistent with myocardial tuberculomas. Those findings are most probably the result of longstanding infection with *M. tuberculosis.*

Other clues to cause of death were provided by sculptor Auguste Clésinger, who shortly after Chopin’s death made both a death mask and several casts of his left hand. The bronzes made from the hand casts do not show clubbed fingers—thickening of the distal phalanges caused by hypoxia, characteristic of pulmonary cystic fibrosis. No other physical evidence supports or excludes any potential diagnosis for Chopin other than tuberculosis, a disease for which there was no effective pharmacologic treatment until 1944, when Albert Schatz, Elizabeth Bugie, and Selman Waksman identified streptomycin with bactericidal activity against mycobacteria.
